# Effects of Soil Salinity on Sucrose Metabolism in Cotton Leaves

**DOI:** 10.1371/journal.pone.0156241

**Published:** 2016-05-26

**Authors:** Jun Peng, Jingran Liu, Lei Zhang, Junyu Luo, Helin Dong, Yan Ma, Xinhua Zhao, Binglin Chen, Ning Sui, Zhiguo Zhou, Yali Meng

**Affiliations:** 1 Key Laboratory of Crop Physiology & Ecology in Southern China, Ministry of Agriculture, Nanjing Agricultural University, Nanjing, China; 2 State Key Laboratory of Cotton Biology, Institute of Cotton Research, Chinese Academy of Agricultural Sciences, Anyang, China; 3 Institute of Integrative Plant Biology, School of Life Science, Jiangsu Normal University, Xuzhou, China; 4 Zhejiang Chinese Medical University, Hangzhou, China; National Key Laboratory of Crop Genetic Improvement, CHINA

## Abstract

This study investigated sucrose metabolism of the youngest fully expanded main-stem leaf (MSL) and the subtending leaf of cotton (*Gossypium hirsutum* L.) boll (LSCB) of salt-tolerant (CCRI-79) and salt-sensitive (Simian 3) cultivars and its relationship to boll weight under low, medium and high soil salinity stress in Dafeng, China, in 2013 and 2014. The results showed that with increased soil salinity, 1) both the chlorophyll content and net photosynthetic rate (*Pn*) decreased, while the internal CO_2_ concentration firstly declined, and then increased in the MSL and LSCB; 2) carbohydrate contents in the MSL reduced significantly, while sucrose and starch contents in the LSCB increased, as did the activities of sucrose phosphate synthase (SPS) and sucrose synthase (SuSy) in both the MSL and LSCB; 3) but invertase activity in both the MSL and LSCB did not change significantly. Our study also showed that the LSCB was more sensitive to soil salinity than was the MSL. Of the measured physiological indices, higher SPS activity, mainly controlled by *sps*3, may contribute to adaption of the LSCB to soil salinity stress because SPS is beneficial for efficiently sucrose synthesis, reduction of cellular osmotic potential and combined actions of *Pn*, and sucrose transformation rate and SPS may contribute to the reduction in boll weight under soil salinity stress.

## Introduction

Soil salinity is a major factor limiting agricultural productivity of nearly 20% of the cultivated area and half of the irrigated area worldwide [1, 2]. Cotton (*Gossypium hirsutum* L.) is a salt-tolerant crop that can improve productivity on saline soil and lead to economic development in regions of high salinity. So far, most studies on halophytes have focused on their morphology, photosynthesis, antioxidant protection system and the ion contents in the soil and in cotton plants [3–5]. However, soil salinity stress inhibits plant growth, mainly by inhibiting leaf expansion and reducing net photosynthetic rate (*Pn*) [6]. The decreased *Pn* is mainly due to stomatal limitation [7] or feedback from increased sucrose in source leaves [8]. The large differences between Rupali (sensitive) and Genesis836 (tolerant) include the salt-induced reduction in net photosynthesis via non-stomatal limitations and damage to photosystem II [9]. In salt-acclimated plants, primary metabolites from amino acid and carbohydrate metabolism are increased and these solutes play a role in osmotic adjustment, membrane and protein protection, or scavenging of reactive oxygen species (ROS) [10]. Analyses of the salt-tolerance of different tomato cultivars found that salt tolerance is regulated mainly by carbon allocation and partitioning, rather than by carbohydrate availability and *Pn* [11].

Sucrose and starch are the principal end-products of photosynthesis in most plants, including cotton. Moreover, as the principal carbohydrate translocated from source to sink tissues [12], sucrose is sensitive to abiotic stress. Sucrose phosphate synthase (SPS), which catalyzes the last step of cytoplasmic sucrose synthesis, is a key regulatory enzyme in carbon partitioning between sucrose and starch in leaves [13] and is often closely correlated with the rate of sucrose export in source tissues [14]. Sucrose synthase (SuSy) plays a crucial role in sucrose breakdown and energy provision [15]. Both enzymes are affected by soil salinity, but their responses to soil salinity vary in different plants and organs. Under soil salinity stress, SPS activity may increase [6] or decline [16, 17], and SuSy activity increases in some species [18].

In cotton, approximately 60%–87% of the carbon in mature bolls comes from CO_2_ assimilation during boll development, in which the subtending leaf of the cotton boll (LSCB) is the most important contributor to the biomass accumulation in seed cotton [19–21], and the youngest fully expanded main-stem leaf (MSL) plays an important role in cotton growth and yield. Most previous studies have mainly focused on changes in the carbohydrate content or sucrose-metabolizing enzymes in the leaves of rice (*Oryza sativa*), tomato (*Lycopersicon esculentum* Mill. and *Solanum lycopersicum*), and other crops [6, 11, 18, 22]. Only Desingh et al. found that Rubisco and SPS activities in cotton declined with increased salinity (mixed salts: NaCl, MgSO_4_ and CaCl_2_) [16]. However, little is known about sucrose metabolism in cotton leaves (both the MSL and LSCB) and its relationship with boll weight under soil salinity stress. The objective of this research, therefore, was to investigate the physiological mechanism for changes in seed fiber weight per boll under soil salinity stress based on sucrose metabolism in the MSL and LSCB.

## Materials and Methods

### Experimental design and treatments

The field experiments were conducted using two cotton cultivars with significant differences in salt tolerance, CCRI-79 (salt-tolerant) and Simian 3 (salt-sensitive), in the state-owned Dafeng Basic Seed Farm in Dafeng (33°20'N, 120°46'E), Jiangsu in 2013 and 2014. No specific permissions were required for using the land in the present study. In addition, the field studies did not involve endangered and protected species. Three fields with similar soil texture and nutrients but low soil salinity (1.15 dS m^-1^, LS), medium soil salinity (MS, 6.00 dS m^-1^) and high soil salinity (11.46 dS m^-1^, HS) were selected, respectively. The LS field had been planted with rice for 5 yrs, the MS field had been planted with rice for 2 yrs, and the HS field had not been planted with rice and cotton before the experiment.

Cotton seeds were directly sown in the field at a density of 45,000 hm^-2^ on 28 April, 2013 and 4 May, 2014. Three replications for each treatment were assigned randomly. Furrow-irrigation was applied as needed to minimize the moisture stress during each season. Conventional weed and insect control measures were utilized as needed.

### Harvest of samples

The MSL were collected once following cotton growth and development period. In addition, white flowers on the first position of the 6–7^th^ fruiting branches of all plants were simultaneously labeled with small plastic tags to verify the uniform metabolic and developmental ages of flowers. These labeled subtending leaves were collected at 9:00–10:00 am once every 7 days from 10 days post-anthesis (DPA) until the boll opening date; they were immediately placed in an ice-box and transported to the laboratory for analysis. The leaves were washed with distilled water and cut along the main vein into two halves. One half was placed immediately in liquid nitrogen and stored at –80°C for subsequent measurements of enzyme and Chl content. The other half was dried at 105°C for 30 min and then at 70°C to constant weight and used for carbohydrate content measurement.

### Photosynthetic pigments and photosynthesis

Chlorophyll (Chl) was extracted from fresh leaf tissue treated with 25 mL of ethanol/acetone (1/1, v/v) for 24 h in the dark. Chl components were then quantified spectrophotometrically at 663 and 645 nm [23].

Photosynthetic parameters of the labeled leaves oriented perpendicular to the sun were measured using an Li-6400 portable photosynthesis system (Li-COR Inc., NE, USA) at light intensity of 1500 μmol m^-2^ s^-1^, relative humidity of (65±5)%, leaf temperature of (32±2)°C and CO_2_ concentration of 380 μmol mol^-1^ from 9:30–11:00 am.

### Carbohydrate content

Leaf tissues were placed in a 10-mL centrifuge tube and mixed with 5 mL of 80% ethanol. The mixture was incubated in a water bath with shaking at 80°C for 30 min and centrifuged at 4000 rpm for 5 min to collect the supernatants. The pellets were subjected to two more extractions using 80% ethanol. All supernatants were combined and diluted to 25 mL with 80% ethanol, mixed, and stored at –20°C for measuring soluble sugar and sucrose.

The ethanol-insoluble residue was subjected to starch extraction. After ethanol was evaporated, starch in the residue was dissolved in 2 mL of distilled water by boiling for 15 min and cooled to room temperature. The leaf starch was then hydrolyzed with 9.2 mol L^-1^ HClO_4_ (2 mL) for 15 min, diluted with 4 mL of distilled water, and centrifuged at 4000 rpm for 10 min. The pellet was extracted one more time using 4.6 mol L^-1^ HClO_4_ (2 mL). The supernatants were retained, combined, and diluted with distilled water to 25 mL.

Soluble sugar and starch contents in the collected extracts were determined using the anthrone method [24]. Sucrose content was assayed in the resuspended supernatant according to previously described protocols [25].

### Measurement of sucrose-metabolizing enzymes

The enzymes SPS and SuSy were extracted from frozen leaf samples as previously described [26], and their activities were measured in 550 μL of 50 mM 6-diphosphate (for SPS) or 50 mM D-fructose (for SuSy), 50 mM UDP-glucose, 10 mM MgCl_2_, 50 mM extraction buffer,and 200 μL of extract. The reaction was conducted at 30°C for 30 min and stopped by adding 100 μL of 2 mol L^-1^ of NaOH and heating for 10 min at 100°C to destroy unreacted hexoses and hexose phosphates. Then, the solution was cooled and mixed with 1 mL of 0.1% (w/v) resorcin in 95% (v/v) ethanol and 3.5 mL of 30% (w/v) HCl before being incubated for 10 min at 80°C. Sucrose content was calculated from a standard curve measured at *A*_*480*_ nm.

Invertase (Inv) was extracted using 0.1 M of phosphate-citrate buffer (pH 5.0) and was measured in a reaction solution containing 1 mol L^-1^ sucrose and the enzyme solution. After incubation at 30°C for 1 h, the reaction solution was mixed with 1 ml of DNS and boiled in a water bath for 5 min. The absorbance at 540 nm was measured using the inactivated enzyme solution as the control, which was used to calculate Inv activity [27].

### RNA extraction, cDNA preparation and quantitative RT-PCR

Total RNA of the LSCB was isolated using the CTAB-sour phenol extraction method [28] with some modifications. RNA samples were treated with DNase I (TaKaRa) after the extraction to eliminate the trace contaminants of genomic DNA. Premier 6.0 and Beacon designer 7.8 were used to design PCR primer design and synthesis. Approximately two micrograms of DNA-free total RNA from each sample was used to synthesize first-strand cDNA in a 20 μl reaction solution using an M-MLV reverse transcription kit (TaKaRa), and the synthesized cDNAs were used as templates in the following qRT-PCR reactions. For each target gene, PCR amplification was performed with specific primer pairs (Table 1). The reaction was conducted using the SYBER premix ExTaq kit (TaKaRa) according to the Manufacturer's instructions. PCR amplification of 18S rRNA was performed for normalization between treated and control samples.

**Table 1:** Primers for real-time RT-PCR amplification and lengths of amplified DNA.

Gene name	Accession number	Forward primer (5’→3’)	Reverse primer (5’→3’)	Length of amplified DNA /bp
*sps*1	*Ghi*.18425	CCTGCCTCTTTCATGTTCAA	CAGTCCTACTCGCTACTTCGTG	226
*sps*2	*Ghi*.24500	GCGTTTAGCCTTTCTTTGAG	ATTTCGTGGAGGAGGTCATT	188
*sps*3	*Ghi*.15278	CAGTCCAACTCGTTACTTCGTG	CCCCTCAAGCTGCTTCTTCT	152
*Susy*A	U73588	GACAAGATGAAATACAAAGGAGC	CATTGGGCCGGTTTTTCTTGGAG	62
*Susy*B	JN376125	GGCTTTTTCTTGTCCGACCATA	AAAGGAAGAGGCGGGTTTTCC	105
*Susy*C	JN376126	CAATGGGACCAAACCCAGAGTTC	AGCAAAAGGCTGCTTGGAAAC	150
*Susy*D	JN376127	CAAGACAGACTCATCTTACTGGACCA	ATGTTGAAACAATGCCCAAAACATGAA	384
18 S rRNA	L24145	TGACGGAGAATTAGGGTTCGA	CCGTGTCAGGATTGGGTAATTT	100

### Weather data

**Fig 1:**
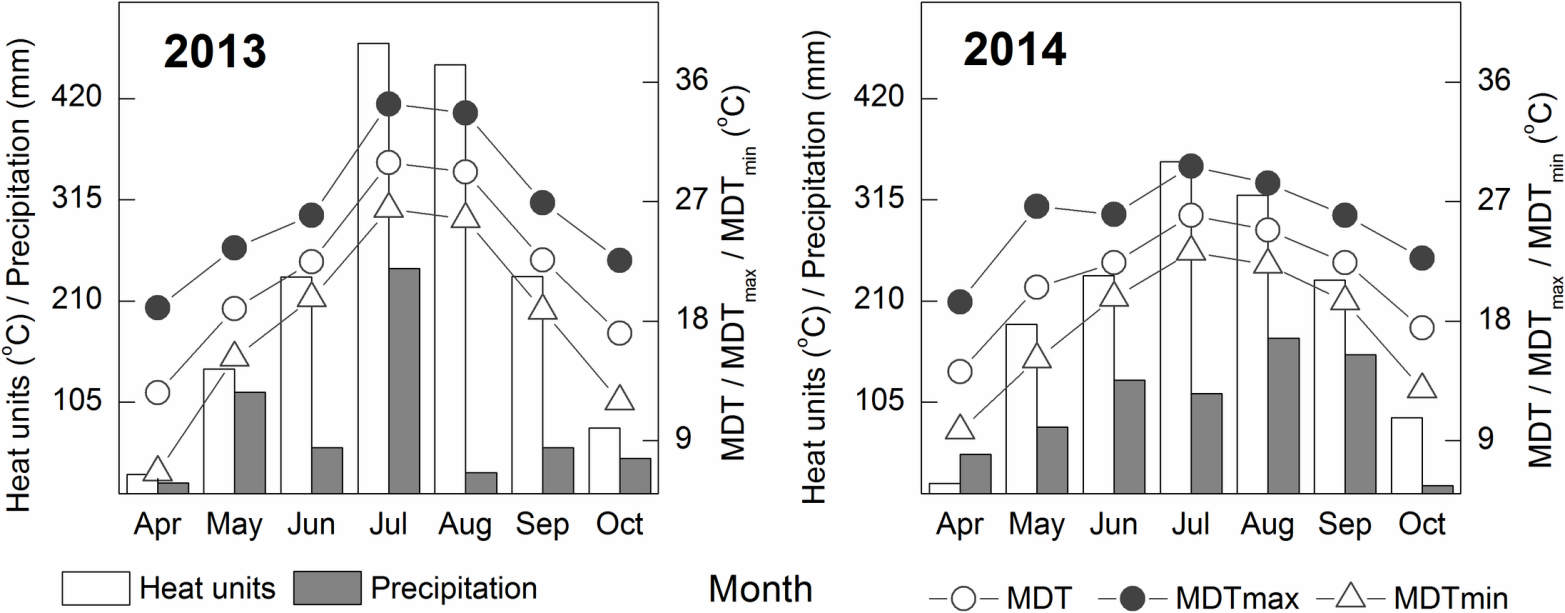
Mean monthly temperature (mean, maximum and minimum), heat units and precipitation during cotton growth period in 2013 to 2014. Heat units were calculated from maximum and minimum temperatures as ∑[(max. temp. + min. temp.)/2–15°C].

The monthly weather data consisting of mean daily temperature (MDT), mean daily maximum temperature (MDTmax), mean daily minimum temperature (MDTmin), total heat units, and precipitation from April to October during cotton growing period for the 2 yrs of this study are given in Fig 1. Total heat units were calculated from maximum and minimum temperatures as ∑[(max. temp. + min. temp.)/2–15°C].

### Data analysis

Data were processed and graphed using Origin 8.1 and subjected to analysis of variance using SPSS (ver. 17.0; SPSS, Chicago, IL, USA) software. The differences between means were identified by LSD (*P*<0.05). The coefficient of variation (CV, %) was calculated as the ratio of the standard deviation to the mean × 100. The maximum sucrose content in the LSCB indicates the amount of available sucrose, while the minimum sucrose content in the LSCB indicates the residual sucrose content. Thus, sucrose transformation rate of the LSCB can be calculated by the expression of 100 × [(maximum sucrose content − minimum sucrose content)/maximum sucrose content].

The comparative CT (△△CT) method was used to analyzed the relative expression levels of isoform genes for *sus* (*sus*A, *sus*B, *sus*C and *sus*D) and *sps* (*sps*1、*sps*2 and *sps*3) for different soil salinities [29]. The control was the untreated samples at 10 DPA under LS. The 2^-△△CT^ value was given to estimate the relative expression of soil salinity materials according to the control at 10, 17, 24 and 31 DPA. The equation was $2^{-\Delta \Delta CT}=2^{-(CT_{reference}-CT_{18\;SrRNA})}/2^{-(CT_{t\arg et}-CT_{18\;SrRNA})}$.

## Results

### Photosynthetic pigments

**Fig 2:**
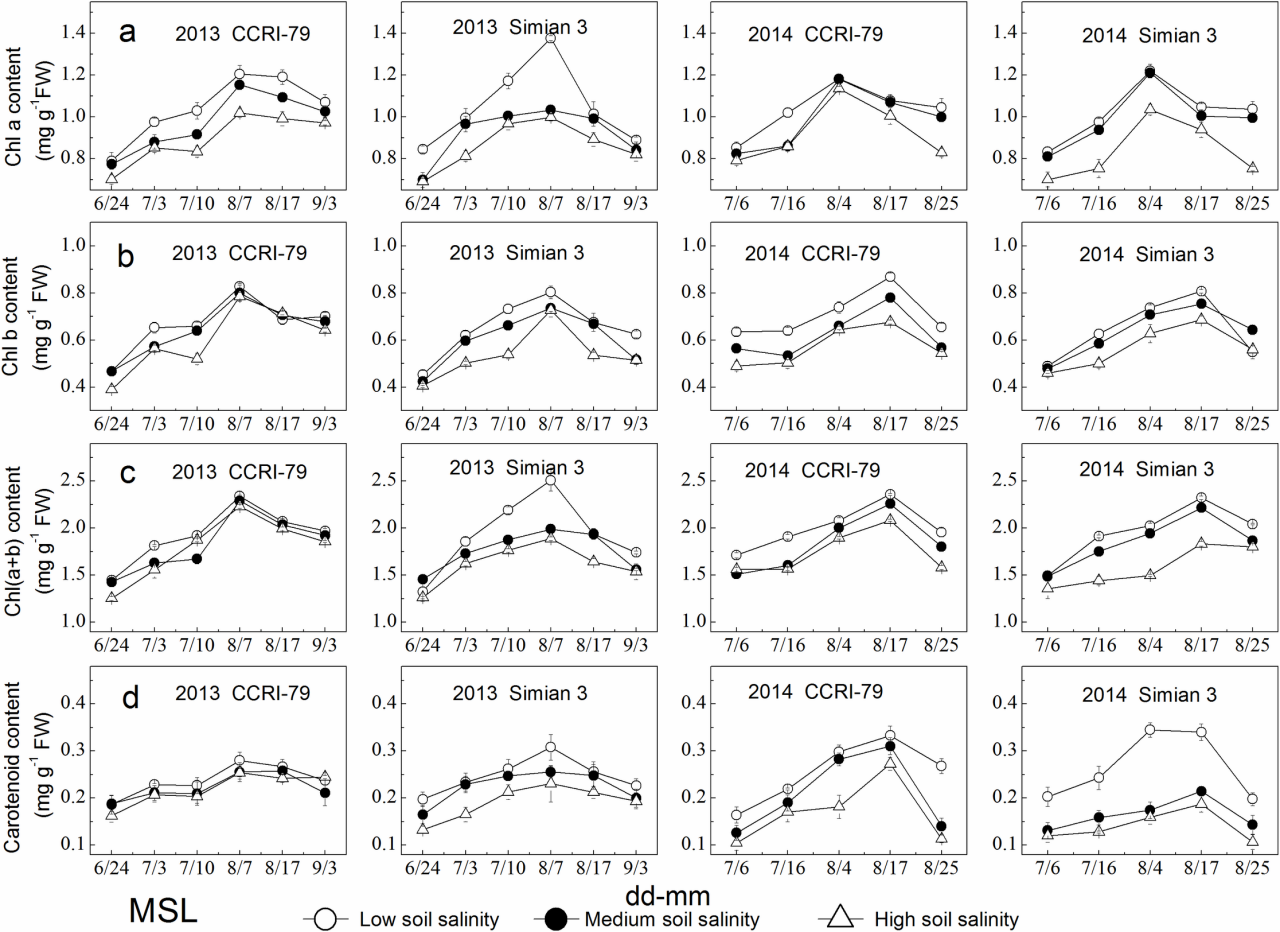
Effects of soil salinity on chlorophyll components in the youngest fully expanded main-stem leaf (MSL). (a) Chl a content. (b) Chl b content. (c) Chl(a+b) content. (d) carotend content.

The contents of Chl components in the MSL of both cultivars changed, showing a single peak trend during cotton growth process, and declined with increased soil salinity (Fig 2). Under soil salinity stress, Chl a content in the MSL decreased by 5%–14% for CCRI-79 and 3%–18% for Simian 3, respectively. The decreases in Chl b and Chl (a+b) were similar to that of Chl a. In addition, carotenoid content decreased by 7%–47% under soil salinity stress, a greater decrease than those of Chl a, Chl b and Chl (a+b).

**Fig 3:**
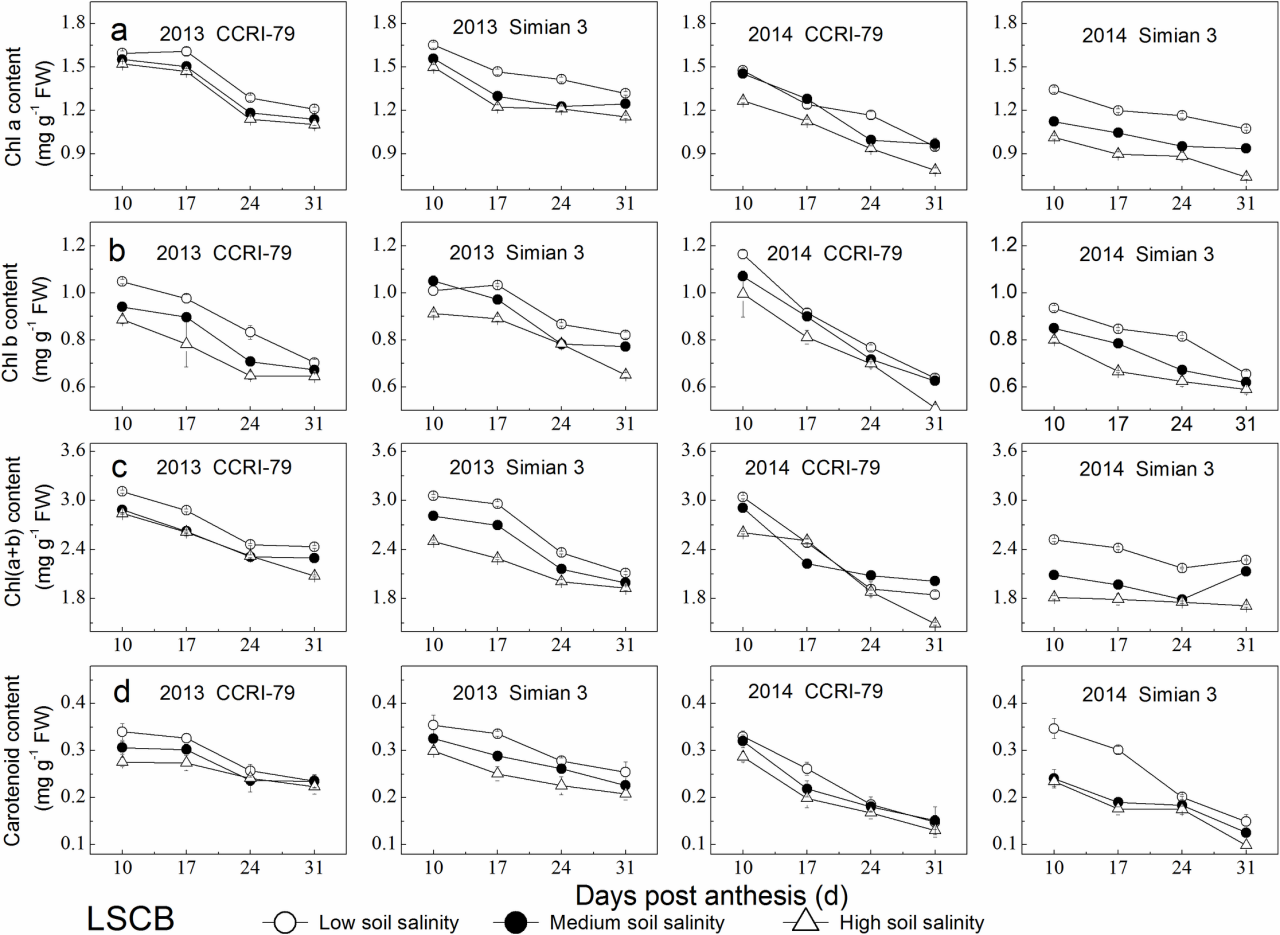
Effects of soil salinity on chlorophyll components in the subtending leaf of cotton boll (LSCB). (a) Chl a content. (b) Chl b content. (c) Chl(a+b) content. (d) Carotend content.

The contents of Chl components in the LSCB decreased with increased DPA and soil salinity (Fig 3). Under soil salinity stress, Chl a, Chl b and Chl(a+b) decreased by 3%–26%, 4%–18%, and 1%–25% for both cultivars, respectively, indicating that Chl a was the most sensitive index in the LSCB to soil salinity stress, and carotenoids content decreased by 6%–32%, greater than the decreases in Chl a, Chl b and Chl(a+b).

### Photosynthetic parameters

**Fig 4:**
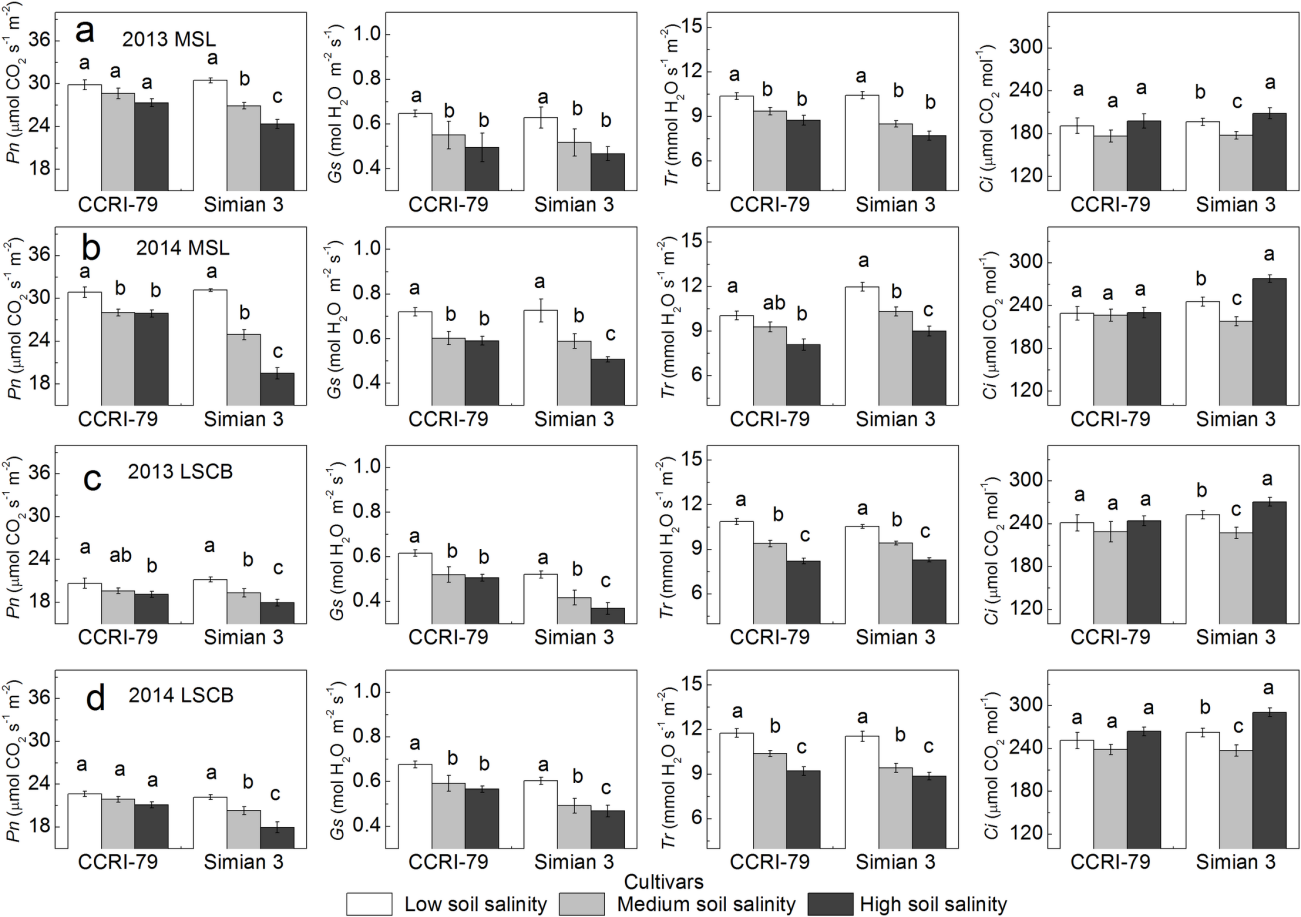
Effects of soil salinity on photosynthetic parameters in the youngest fully expanded main-stem leaf (MSL) and the subtending leaf of cotton boll (LSCB). (a) Photosynthetic parameters of the MSL in 2013. (b) Photosynthetic parameters of the MSL in 2014. (c) Photosynthetic parameters of the LSCB in 2013. (d) Photosynthetic parameters of the LSCB in 2014.

With increased soil salinity, all of *Pn*, stomatal conductance (*Gs*) and transpiration rate (*Tr*) all showed downward trends in the MSL and LSCB, and internal CO_2_ concentration (*Ci*) showed a declining and then rising trend (Fig 4). Under soil salinity stress, *Pn* decreased by 4%–10% for CCRI-79 and 12%–38% for Simian 3 in the MSL, and by 3%–8% for CCRI-79 and 9%–19% for Simian 3 in the LSCB, respectively. *Ci* in the MSL and LSCB declined by about 1%–11% and 5%–10% under medium salinity treatment (MS), respectively, and about 1%–13% and 1%–11% under high soil salinity treatment (HS), respectively. In addition, *Pn* and *Ci* in the MSL and LSCB were significantly different among the three soil salinity levels for Simian 3 (*P*<0.05), but not for CCRI-79.

### Carbohydrate contents

**Fig 5:**
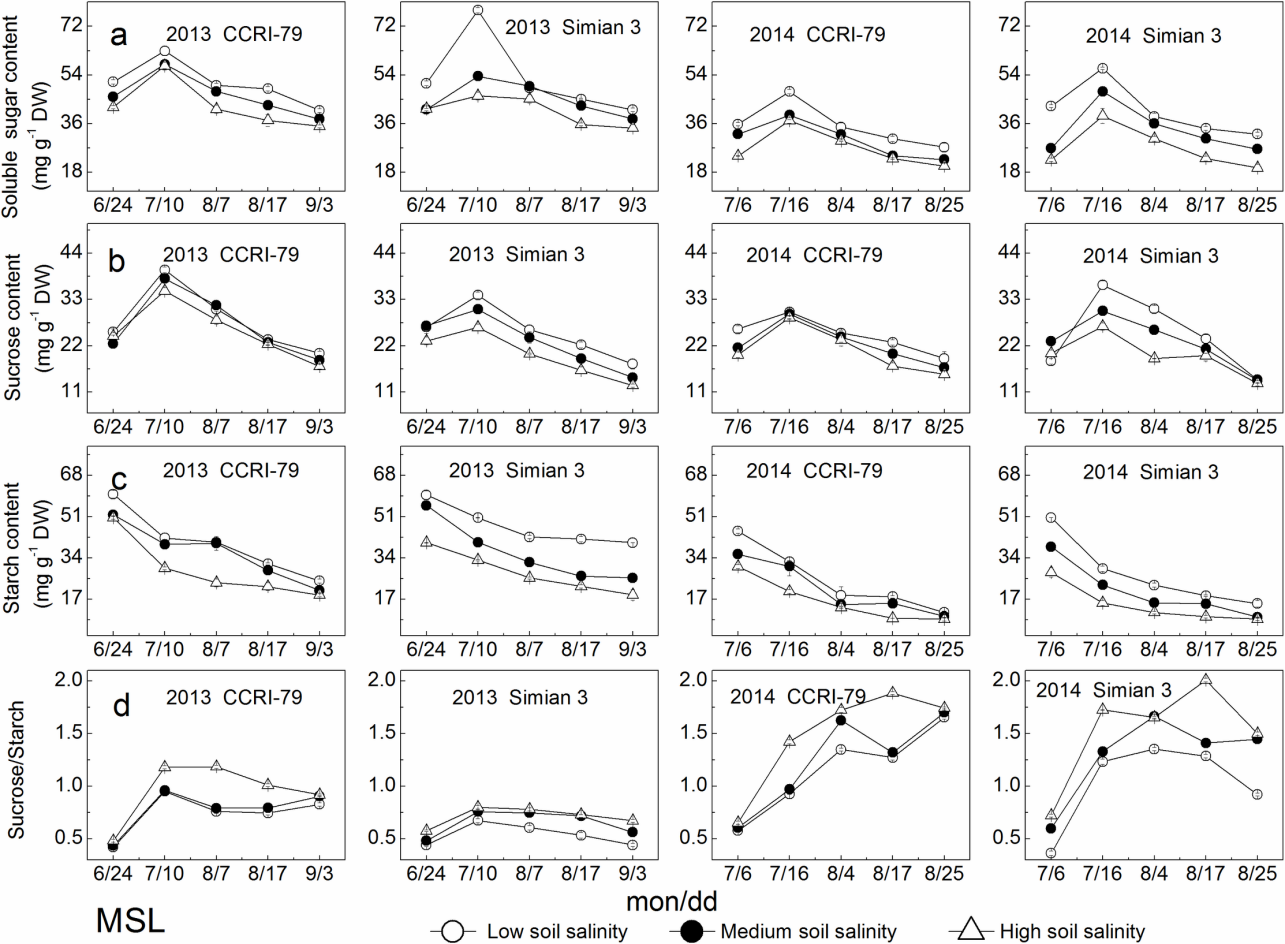
Effects of soil salinity on carbohydrates content in the youngest fully expanded main-stem leaf (MSL). (a) Soluble sugar content. (b) Sucrose content. (c) Starch content. (d) Sucrose/Starch.

During cotton growth process, the contents of soluble sugar and sucrose in the MSL changed showing a single maximum peak in mid-July and declined with increased soil salinity (Fig 5A and 5B). Under soil salinity stress, the contents of soluble sugar, sucrose and starch decreased by 9%–34%, 5%–22% and 9%–46%, respectively, for both cultivars. Starch content in the MSL had a declining trend during cotton growth process and with increased soil salinity, similar to those of soluble sugar and sucrose (Fig 5C). The ratio of sucrose/starch in the MSL first increased, reaching their highest levels from mid-July to mid-August, and then declined with cotton growth progress (Fig 5D) and increased by 5%–48% under soil salinity stress for both cultivars.

**Fig 6:**
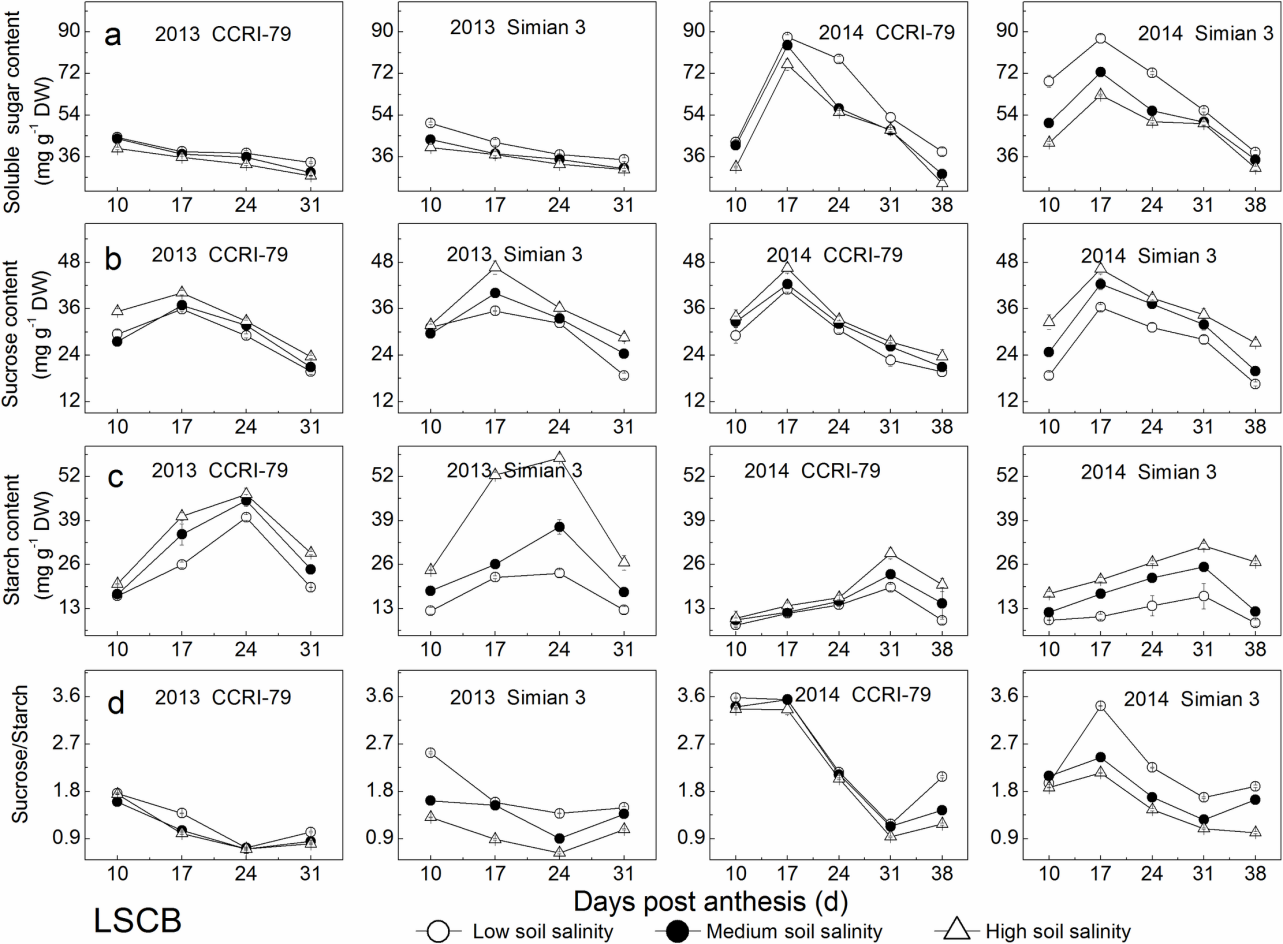
Effects of soil salinity on carbohydrates content in the subtending leaf of cotton boll (LSCB). (a) Soluble sugar content. (b) Sucrose content. (c) Starch content. (d) Sucrose/Starch.

As shown in Fig 6A and 6B, with increased DPA, the contents of soluble sugar and sucrose in the LSCB declined in 2013, and showed a single peak occurring at 17 DPA in 2014. Under soil salinity stress, soluble sugar content was reduced by 5%–26%, and sucrose content increased by 3%–37% for both cultivars. In addition, the increase in sucrose content was greater than the decrease in soluble sugar content. Moreover, the changes in sucrose and soluble sugar for CCRI-79 were significantly lower than those for Simian 3. Under soil salinity stress, the maximum sucrose content in the LSCB significantly increased, and the sucrose transformation rate significantly declined (S1 Table). In addition, the CV of the sucrose transformation rate of Simian 3 was significantly higher than that of CCRI-79. With increased DPA and soil salinity, starch content increased, and the ratio of sucrose/starch declined (Fig 6C and 6D). Under soil salinity stress, starch content increased by 19%–43% for CCRI-79 and 41%–128% for Simian 3, and the ratios of sucrose/starch decreased by 7%–44% for both cultivars, indicating that the change in sucrose content was lower than that in starch content.

### Activities of sucrose metabolic enzymes

#### SPS activities

**Fig 7:**
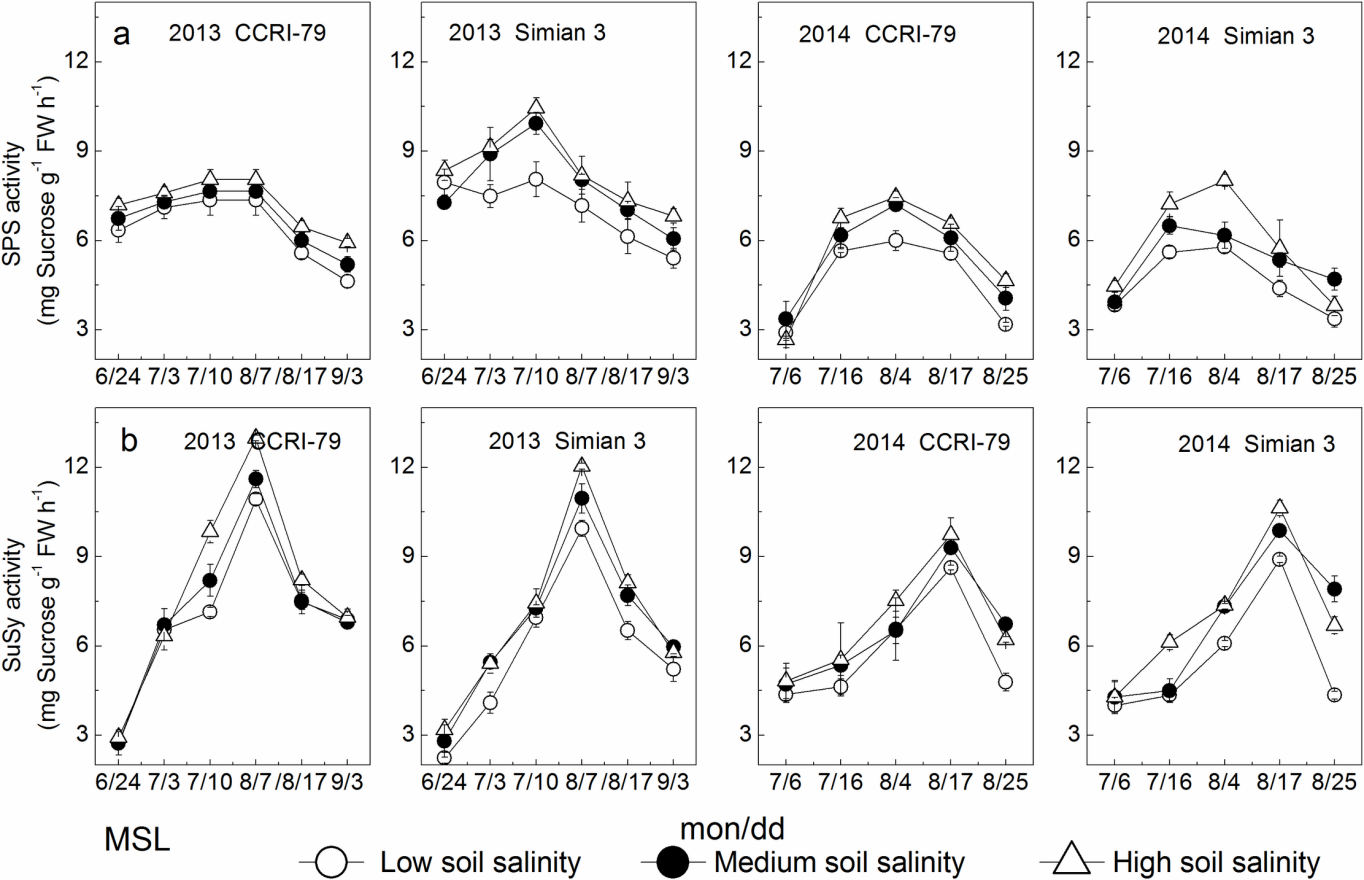
Effects of soil salinity on SPS and SuSy activities in the youngest fully expanded main-stem leaf (MSL). (a) SPS activity. (b) SuSy activity.

SPS activity in the MSL changed with a single peak in mid-August during cotton growth progress (Fig 7A). The peak SPS activity increased with increased soil salinity and significantly differed between the two cultivars among the three soil salinity levels (from LS to MS and/or HS) (*P*<0.05, S2 Table). However, under soil salinity stress, SPS activity increased by only 5%–10% in CCRI-79, significantly lower than that of 12%–39% in Simian 3. Analysis of variance also found that SPS peak activity in the MSL showed significant differences between years as well as cultivars, and among salinity levels, and the interaction between cultivars and soil salinity levels had significant effects on SPS activity (*P*<0.05, Table 2).

**Table 2:** *F* values of ANOVA of the effect of year, salinity level, cultivar, and their interactions with sucrose phosphate synthase (SPS) and sucrose synthase (SuSy) peak values in 2013 and 2014. MSL: the youngest fully expanded main-stem leaf, LSCB: the subtending leaf of cotton boll.

Items	Year (Y)	Cultivar (C)	Salinity level (S)	Y × C	Y × S	C × S	Y × C × S
MSL							
SPS	200.0**	37.6**	56.4**	90.6**	2.1ns	17.1**	3.1ns
SuSy	428.9**	2.3ns	121.1**	62.3**	5.5*	1.3ns	1.2ns
LSCB							
SPS	105.3**	81.1**	230.0**	37.5**	0.5ns	66.4**	5.4*
SuSy	117.9**	39.1**	121.0**	291.3**	6.2**	0.4ns	2.7ns

* and ** indicate significant differences at 0.05 and 0.01 probability levels, respectively; ns, not significant.

**Fig 8:**
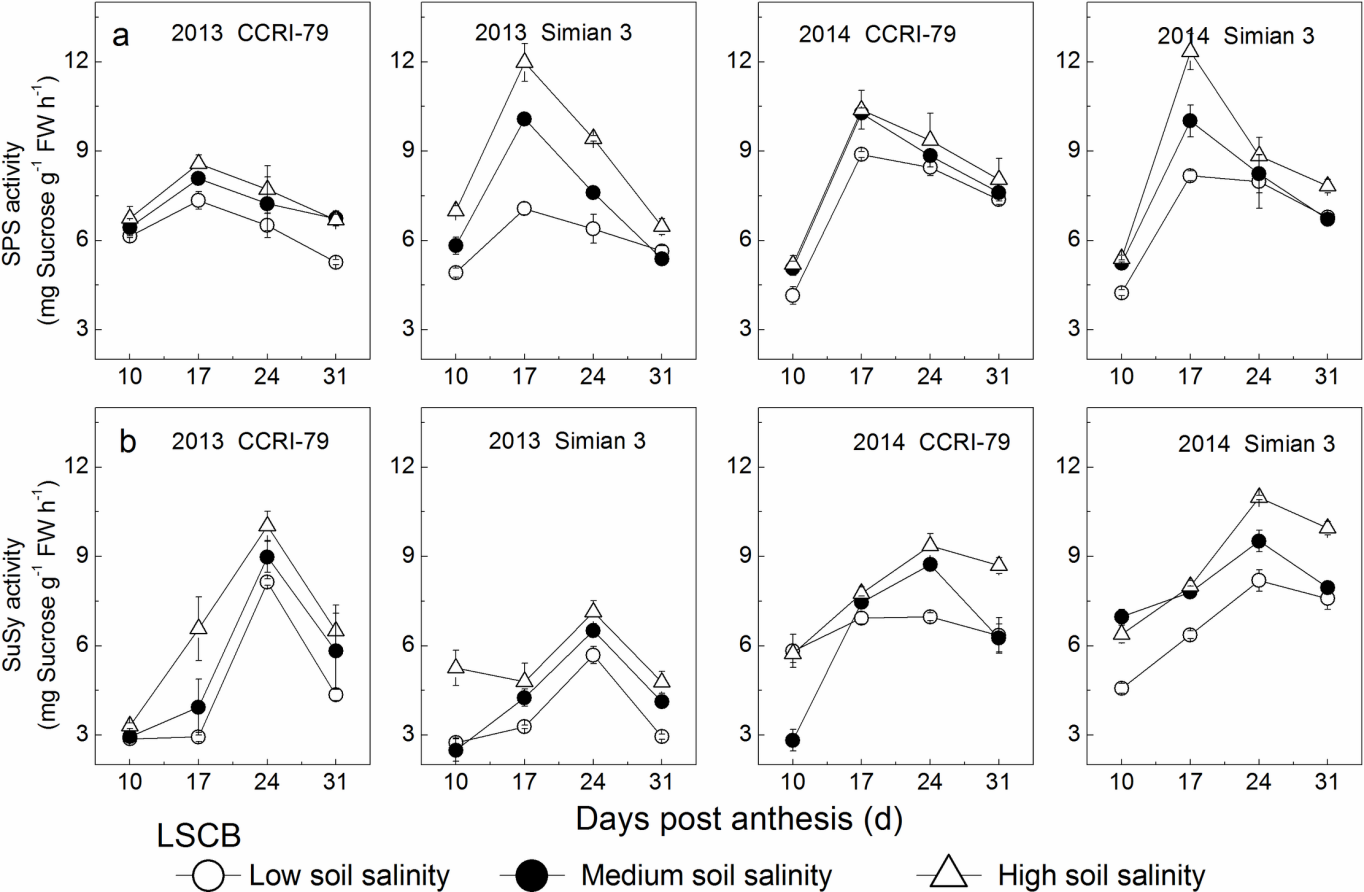
Effects of soil salinity on SPS and SuSy activities in the subtending leaf of cotton boll (LSCB). (a) SPS activity. (b) SuSy activity.

SPS activity in the LSCB increased from 10 DPA, reached its highest level at 17 DPA, and then declined significantly (Fig 8A). The peak SPS activity increased with the increase of soil salinity and significantly differed between CCRI-79 and Simian 3 among the three soil salinity levels (from LS to MS and/or HS) (*P*<0.05, S2 Table). However, under soil salinity stress, SPS activity increased only by 10%–17% in CCRI-79, significantly lower than in Simian 3 (23%–70%). Moreover, peak SPS activity in the LSCB significantly differed between years as well as cultivars, and among salinity levels, and the interactions between cultivars and soil salinity levels had significant effects on SPS activity (*P*<0.05, Table 2).

#### SuSy activities

SuSy plays an important role in sucrose degradation in vivo. The changes in SuSy activity in the MSL and LSCB were similar to those of SPS activity (Figs 7B and 8B). However, no significant differences were observed between the two cultivars among the three soil salinity levels (*P*>0.05, Table 2).

#### Inv activity

**Fig 9:**
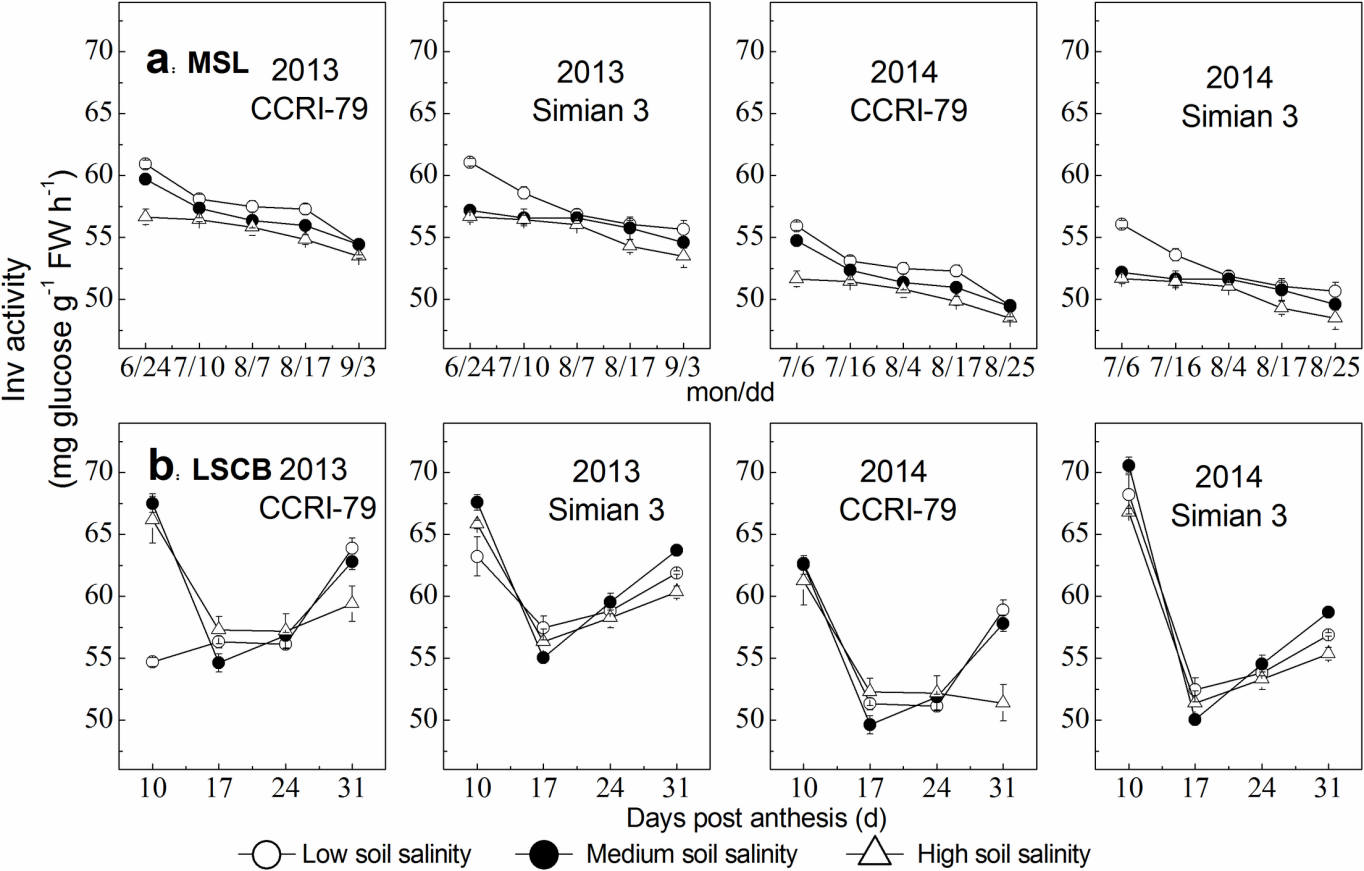
Effects of soil salinity on Inv activity in the youngest fully expanded main-stem leaf (MSL) and the subtending leaf of cotton boll (LSCB). (a) Inv activity in the MSL. (b) Inv activity in the LSCB.

Inv activity declined in the MSL during cotton growth progress, and showed a decline, followed by an increase trend with increased DPA in the LSCB (Fig 9). However, under soil salinity stress, the decreases in Inv activity of the MSL and LSCB were less than 5% among the three soil salinity levels, and there were non-significant differences for both cultivars in the MSL and LSCB (*P*>0.05).

### Correlation between boll weight and *Pn* as well as carbohydrates and sucrose-metabolizing enzymes

#### Relationship between carbohydrates and sucrose-metabolizing enzymes

In the MSL, soluble sugar and sucrose contents were significantly positively correlated with SPS and Inv, and significantly negatively correlated with SuSy (*P*<0.05, Table 3); starch content was significantly positively correlated with Inv, and significantly negatively correlated with SPS and SuSy; the ratio of sucrose/starch was significantly positively correlated with SPS and SuSy, and significantly negatively correlated with Inv.

**Table 3:** Relationship between carbohydrate content and sucrose metabolism enzymes in the youngest fully expanded main-stem leaf (MSL) and the subtending leaf of cotton boll (LSCB) for cultivars CCRI-79 and Simian 3 for three salinity levels in 2013 and 2014. MSL: the youngest fully expanded main-stem leaf, LSCB: the subtending leaf of cotton boll.

Leaf position	Indicators	Years	Soluble sugar	Sucrose	Starch	Sucrose/Starch
MSL n = 30	SPS	2013	0.387*	0.478**	0.220	0.137
		2014	0.259	0.396*	-0.378*	0.574**
	SuSy	2013	0.104	0.215	-0.537**	0.704**
		2014	-0.426*	-0.279	-0.653**	0.660**
	Inv	2013	0.632**	0.550**	0.875**	-0.408*
		2014	0.627**	0.511**	0.900**	-0.749**
LSCB n = 24	SPS	2013	-0.173	0.798**	0.738**	-0.423*
		2014	0.554**	0.736**	0.318	-0.008
	SuSy	2013	-0.534**	0.085	0.753**	-0.791**
		2014	0.088	0.353	0.673**	-0.463*
	Inv	2013	0.163	-0.483*	-0.545**	0.365
		2014	-0.549**	-0.634**	-0.286	-0.062

* and ** indicate significant differences at 0.05 and 0.01 probability levels, respectively; n = 24, *R*^2^_*0*.*05*_ = 0.404, *R*^2^_*0*.*01*_ = 0.515; n = 30, *R*^2^_*0*.*05*_ = 0.361, *R*^2^_*0*.*01*_ = 0.463. LS: low soil salinity, MS: medium salinity, HS: high soil salinity.

In the LSCB, soluble sugar content was significantly positively correlated with SPS and significantly negatively correlated with SuSy; sucrose content was significantly positively correlated with SPS and significantly negatively correlated with Inv; starch content was significantly positively correlated with SPS and SuSy; and the ratio of sucrose/starch was significantly negatively correlated with SPS and SuSy (*P*<0.05).

#### Relationship among *Pn*, Chl content and sucrose-metabolizing enzymes

In the MSL and LSCB, *Pn* was significantly positively correlated with Chl components as well as carotenoid, and significantly negatively correlated with SPS and SuSy in both years (*P*<0.05, Table 4).

**Table 4:** Relationship between photosynthetic rate (*Pn*) and Chl and sucrose-metabolizing enzymes in the youngest fully expanded main-stem leaf (MSL) and the subtending leaf of cotton boll (LSCB) for cultivars CCRI-79 and Simian 3 for three salinity levels in 2013 and 2014.

Years	Chl a	Chl b	Chl(a+b)	Car	SPS	SuSy
MSL						
2013	0.958**	0.969**	0.991**	0.965**	-0.881*	-0.481
2014	0.861*	0.654	0.887*	0.882*	-0.832*	-0.839*
LSCB						
2013	0.987**	0.675	0.926**	0.910*	-0.987**	-0.664
2014	0.966**	0.947**	0.978**	0.901*	-0.732	-0.921**

* and ** indicate significant differences at 0.05 and 0.01 probability levels, respectively; n = 6, *R*^2^_*0*.*05*_ = 0.811, *R*^2^_*0*.*01*_ = 0.917.

#### Correlation analyses between boll weight and *Pn*, carbohydrate contents and sucrose-metabolizing enzymes

In the MSL, boll weight was positively correlated with *Pn* and carbohydrate contents, and negatively correlated with the sucrose/starch ratio and SuSy (*P*<0.05, Table 5). In the LSCB, boll weight was positively correlated with *Pn* and soluble sugar content and negatively correlated with sucrose, starch, SPS and SuSy (*P*<0.05).

**Table 5:** Correlation coefficients between boll weight and photosynthetic rate (*Pn*) carbohydrate contents, and sucrose-metabolizing enzymes in the youngest fully expanded main-stem leaf (MSL) and the subtending leaf of cotton boll (LSCB) for cultivars CCRI-79 and Simian 3 for three salinity levels in 2013 and 2014.

Years	*Pn*	Soluble sugar	Sucrose	Starch	Sucrose/Starch	SPS	SuSy
MSL							
2013	0.884*	0.928**	0.766	0.816*	-0.358	-0.833*	-0.558
2014	0.883*	0.963**	0.963**	0.957**	-0.921**	-0.957**	-0.949**
LSCB							
2013	0.905*	0.791	-0.888*	-0.742	0.494	-0.880*	-0.623
2014	0.877*	0.919**	-0.946**	-0.909*	0.702	-0.904*	-0.845*

* and ** indicate significant differences at 0.05 and 0.01 probability levels, respectively; n = 6, *R*^2^_*0*.*05*_ = 0.811, *R*^2^_*0*.*01*_ = 0.917.

### Gene expression in the LSCB under soil salinity stress

#### Relative expression levels of *sps* isozyme genes

**Fig 10:**
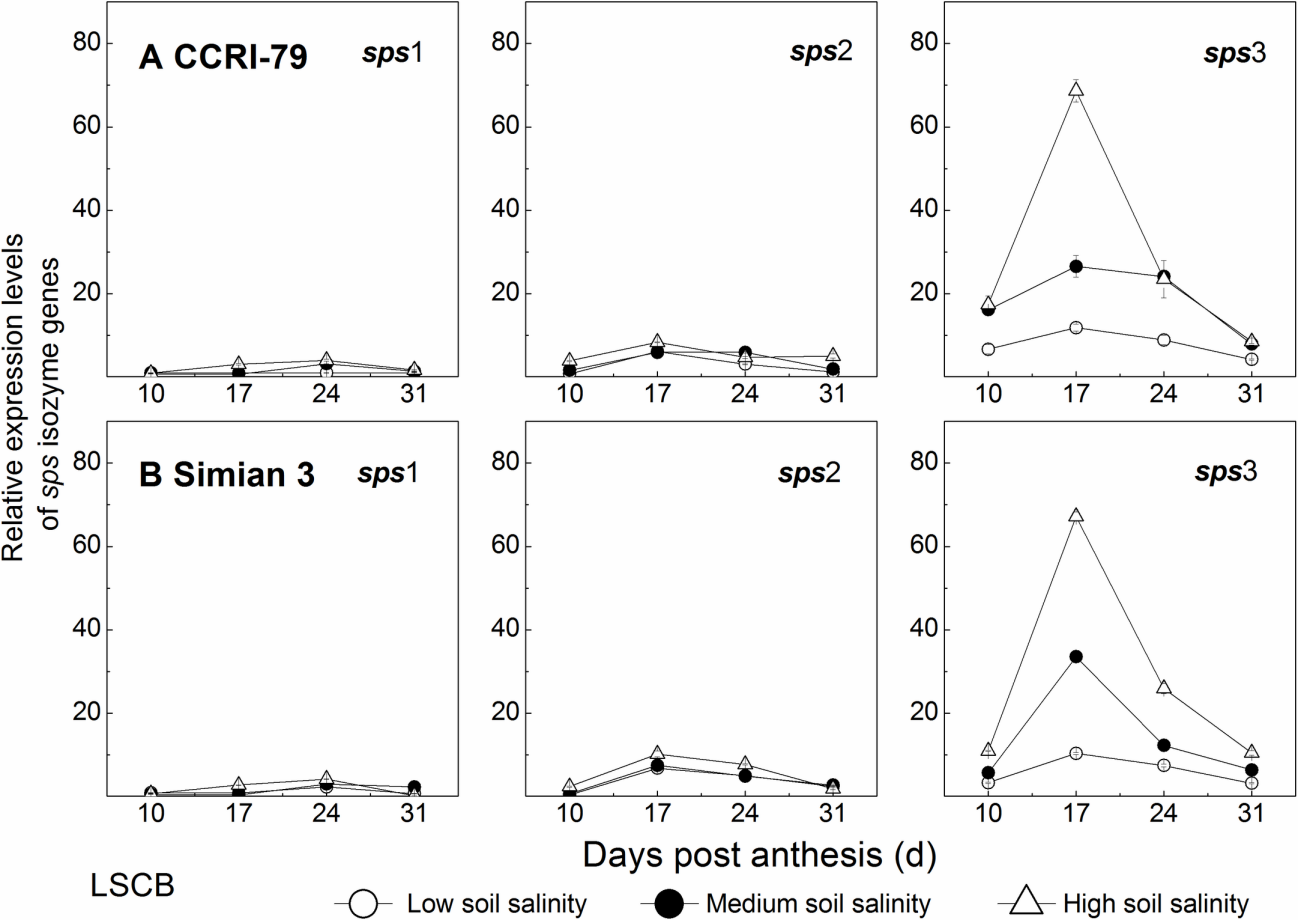
Effects of soil salinity on relative expression levels of *sps* isozyme genes in the subtending leaf of cotton boll (LSCB). (A) Relative expression levels of *sps* isozyme genes for CCRI-79. (B) Relative expression levels of *sps* isozyme genes for Simian 3.

In the LSCB, there were significant differences in relative expression levels among the *sps* isoforms as shown in Fig 10. In particular, the relative expression level of *sps*3 was higher than those of *sps*1 and *sps*2 during boll development. With the mean of all data in each of the three *sps* isoforms, the proportion of *sps*3 expression levels was approximately 75.0%, which was much higher than those of *sps*1 and *sps*2. This result indicated that *sps*3 was the major isoform gene in sucrose metabolism. The relative expression levels of each *sps* isoform changed with a single peak at 17 DPA during boll development (Fig 10). Under soil salinity, the relative expression level of *sps*3 in the LSCB increased remarkably, especially at 17 DPA.

In this study, the relative expression levels of *sps*3 isoform in CCRI-79 and Simian 3 increased significantly by 1.4–2.7 and 1.4–3.7 fold, respectively. For *sps*1 and *sps*2 isoforms, the increased degrees under soil salinity were less than 1.4 fold in both cultivars.

In general, the difference between CCRI-79 (tolerant to soil salinity) and Simian 3 (sensitive to soil salinity) in the LSCB was mainly affected by *sps*3 modulated changes in SPS activities under soil salinity.

#### Relative expression levels of *sus* isozyme genes

**Fig 11:**
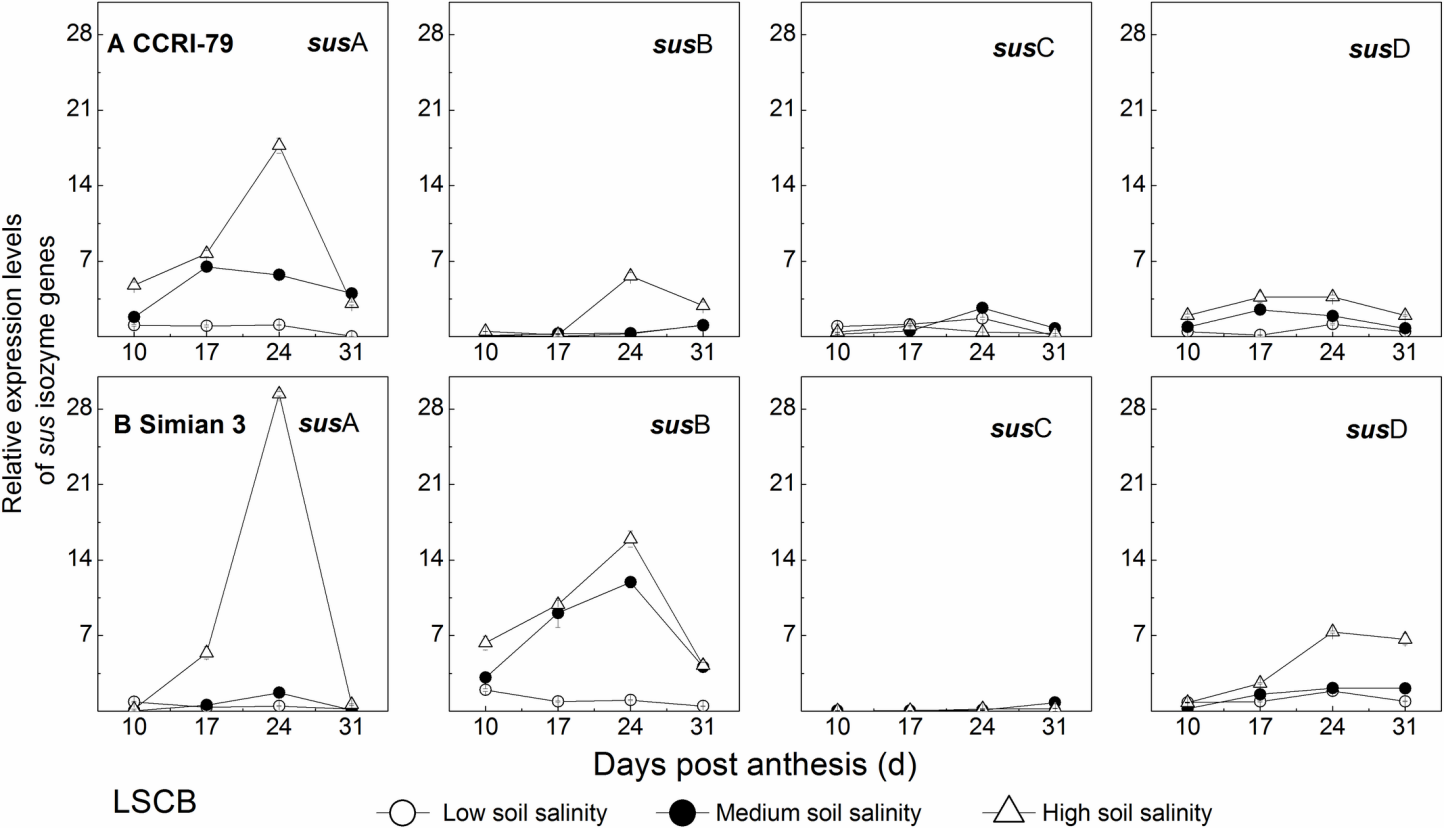
Effects of soil salinity on relative expression levels of *sus* isozyme genes in the subtending leaf of cotton boll (LSCB). (A) Relative expression levels of *sus* isozyme genes for CCRI-79. (B) Relative expression levels of *sus* isozyme genes for Simian 3.

In the LSCB, there were significant differences in relative expression levels among the *sus* isoforms as shown in Fig 11. In particular, the relative expression levels of *sus*A and *sus*B were higher than those of *sus*C and *sus*D during boll development. With the mean of all data in each of the four *sus* isoforms, the proportions of *sus*A and *sus*B expression levels were approximately 40.1% and 34.6%, respectively, which were much higher than those of *sus*C and *sus*D. This result indicated that *sus*A and *sus*B were the major isoform genes. The relative expression levels of each *sus* isoform changed with a single peak at 24 DPA during boll development (Fig 11). Under soil salinity, the relative expression levels of *sus*A and *sus*B in the LSCB increased remarkably, especially at 24 DPA.

In this study, for *sus*A isoform, the relative expression levels of CCRI-79 and Simian 3 increased significantly by 4.6–9.3 and 0.3–18.0 fold during boll development, respectively; for *sus*B isoform, the increased degrees for both cultivars were about 0.2–5.0 and 5.5–7.4 fold, respectively; for *sus*C and *sus*D isoforms, the increased degrees under soil salinity were less than 2.3 fold in both cultivars.

## Discussion

The youngest fully expanded main-stem leaf (MSL) and subtending leaf of cotton boll (LSCB) are important photosynthetic organs that play pivotal roles during cotton growth. The different leaf positions may lead to differences in their responses to soil salinity. Decrease in Chl content under soil salinity has been reported in rice [6] and maize [7]. Significantly greater damage to Chl a than Chl b has been reported under soil salinity stress [30], consistent with our results. Under soil salinity stress, Chl content decreased and SPS and SuSy activities increased in both the MSL and LSCB, consistent with previous studies on other crops [6, 7, 16]. In addition, these indices changed more in the LSCB than in the MSL, suggesting that the LSCB were more sensitive to soil salinity than the MSL. Stomatal closure was the main reason for the decrease in *Pn* under low salinity stress [31]. However, with increased soil salinity, non-stomatal limitations, such as increase in sucrose-metabolic enzymes activities and Chl degradation, became the major factors limiting photosynthesis [3, 6]. Our study found that in the MSL and LSCB, *Pn* and Chl contents decreased and SPS and SuSy activities increased under MS and HS, whereas *Ci* was reduced under MS, and increased under HS. Our study also showed that in the MSL and LSCB, the decreased *Pn* was due to the co-regulation of both stomatal and non-stomatal factors under MS, but mainly by non-stomatal factors under HS.

Sugars (i.e. starch and sucrose) are primary products of photosynthesis in higher plants [12, 32]. Soluble sugars are highly sensitive to environmental stresses and the major forms of carbohydrates. Sugars not only provide energy and solutes for osmotic adjustment, but also modulate expression of multiple genes as regulatory messengers [33]. Previous studies showed that sucrose translocation from leaf to root was inhibited under salt stress, resulting in an accumulation of carbohydrate in leaf tissue [11]. Soluble sugar and sucrose were relatively constant in the leaf of salt-tolerant varieties, but increased in salt-sensitive varieties [22]. In this study, with increased soil salinity, soluble sugar and starch contents decreased, while the activities of SuSy and SPS and the sucrose/starch ratio increased in the MSL. These results showed that under soil salinity stress, photosynthetic product distribution favored sucrose synthesis in the MSL. Meanwhile, soil salinity promoted sucrose exportation in the MSL, lowering sucrose content. However, further study is needed to determine whether this could eventually increase carbohydrate content in other organs (e.g., stems and roots). Moreover, in the MSL, soluble sugar and sucrose was significantly positively related to SPS activity, and the ratio of sucrose/starch was significantly positively correlated with SPS and SuSy activities, while starch content was significantly negatively correlated with SPS activity, and soluble sugar, sucrose and starch were significantly negatively correlated with SuSy activity (*P*<0.05). Our study also showed that in the MSL, higher SPS activity favored the conversion of photosynthate to sucrose, and higher SuSy activity was beneficial to exporting sucrose, consistent with previous studies [34].

In cotton, 60%–87% of bolls’ dry matter was exported from the LSCB [17–19]. Under soil salinity stress, soluble sugar content decreased, but large amounts of sucrose and starch were accumulated in the LSCB. In addition, the increase in starch content was greater than that of sucrose content. This may be an indication of changing carbon partitioning between sucrose and starch to retain greater amounts of fixed carbon in the LSCB under soil salinity stress. Therefore, under soil salinity stress, sufficient non-structural carbohydrates (such as sucrose and starch) were stored in the LSCB, and sucrose might not be exported efficiently to cotton bolls, consistent with the reduced sucrose transformation rate and *Pn* (S1 Table, Fig 4). These findings might explain the decreased boll weight under soil salinity stress. Furthermore, higher sucrose content in the LSCB could help maintain cellular homeostasis, consistent with previous studies [6].

Correlation analysis revealed that sucrose was positively correlated with SPS, while starch was positively correlated with SuSy, and the ratio of sucrose/starch was negatively correlated with SuSy (*P*<0.05, Table 4) suggesting that the LSCB could regulate sucrose metabolism by SPS and SuSy under soil salinity stress. In the LSCB, the peak value of SPS and SuSy activity (S2 Table) and their gene expressions, especially *sps*3, *sus*A and *sus*B (Figs 10 and 11) were all increased under soil salinity stress. The maximum SPS activity was increased by 10%–70%, and that of SuSy was increased by 10%–34% under the treatments of MS and HS, compared to that under LS treatment. The expressions of *sps*3, *sus*A and *sus*B in the LSCB were up-regulated by 1.4–3.7, 0.3–18.0 and 0.2–7.4 fold, respectively (Figs 10 and 11). However, of the three enzymatic activities in sucrose metabolism, only SPS activity in the LSCB changed significantly between the two cultivars under different soil salinity levels (*P*<0.05, Table 3). In addition, under soil salinity stress, SPS activity increased by 10%-17% for CCRI-79 and 23%-70% for Simian 3. Therefore, the difference in SPS activity mainly regulated by *sps*3 might be the reason for the different salt sensitivity of the two cultivars in sucrose metabolism. This showed that SPS was implicated in the response of sucrose metabolism to soil salinity stress. Higher SPS activity under soil salinity stress is beneficial for efficiently sucrose synthesis, reduction of cellular osmotic potential and adaptation to the changes in external salt stress [35].

Furthermore, boll weight was significantly positively correlated with *Pn*, while significantly negatively correlated with SPS activity (*P*<0.05). Our study also showed that under soil salinity stress, boll weight was affected by both *Pn* and SPS activity co-regulation in the LSCB. With elevated soil salinity, *Pn* and the sucrose transformation rate decreased in the LSCB, resulting in lower boll weight.

## Conclusion

1. The response of photosynthate formation to soil salinity stress was consistent between the MSL and LSCB. Decreased *Pn* in the MSL and LSCB was mainly caused by co-regulation of stomatal and non-stomatal factors under medium salinity stress and by non-stomatal factors including chlorophyll degradation and increased SPS and SuSy activities under high salinity stress.
2. The response of photosynthate transport to soil salinity stress was different in the MSL and LSCB. With increased soil salinity, the carbohydrate contents (soluble sugar, sucrose and starch) decreased in the MSL, while the contents of sucrose and starch increased in the LSCB. Soil salinity stress promoted sucrose export from the MSL, but inhibited sucrose export from the LSCB to cotton bolls. In addition, the LSCB was more sensitive to soil salinity stress than the MSL.
3. The differences in *Pn*, SPS and sucrose transformation rate in the LSCB between the two cultivars under soil salinity stress may partially explain the greater tolerance of CCRI-79 to soil salinity compared to Simian 3.

## Supporting Information

S1 Table The values of maximum sucrose content and sucrose transformation rate in the subtending leaf of cotton boll (LSCB) at different soil salinity levels in 2013 and 2014. LS: low soil-salinity, MS: medium soil-salinity, and HS: high soil-salinity.
(DOC)

S2 Table The peak values of sucrose phosphate synthase (SPS) and sucrose synthase (SuSy) activities in the youngest fully expanded main-stem leaf (MSL) and the subtending leaf of cotton boll (LSCB) under different soil salinity levels in 2013 and 2014. LS: low soil-salinity, MS: medium soil-salinity, and HS: high soil-salinity.
(DOCX)

## Abbreviations

Chl: Chlorophyll
*Ci*: internal CO_2_ concentration
CV(%): coefficient of variance
DPA: days post anthesis
Inv: invertase
LSCB: the subtending leaf of cotton boll
MSL: the youngest fully expanded main-stem leaf
*Pn*: net photosynthetic rate
SPS: sucrose phosphate synthase
SuSy: sucrose synthase
