# Supplementary material for: Effects of Soil Salinity on Sucrose Metabolism in Cotton Leaves
Source: PLoS One. 2016 May 26;11(5):e0156241. doi: 10.1371/journal.pone.0156241 (PMC4881904; doi:10.1371/journal.pone.0156241)
Supplement: S1 Table — LS: low soil-salinity, MS: medium soil-salinity, and HS: high soil-salinity. (DOC) [file pone.0156241.s001.doc]

**S1 Table. The values of maximum sucrose content and sucrose transformation rate in the subtending leaf of cotton boll (LSCB) at different soil salinity levels in 2013 and 2014.**

| Salinity levels | Maximum sucrose content (mg g^-1^ DW) | | Sucrose transformationrate (%) | | Boll weight (g) | |
| --- | --- | --- | --- | --- | --- | --- |
|  | CCRI-79 | Simian 3 | CCRI-79 | Simian 3 | CCRI-79 | Simian 3 |
| 2013 |  |  |  |  |  |  |
| LS | 35.9 c | 35.4 c | 44.9 a | 47.2 a | 4.6 a | 4.4 a |
| MS | 36.9 b | 40.0 b | 43.4 ab | 39.1 b | 4.2 ab | 4.1 ab |
| HS | 40.2 a | 46.7 a | 41.3 b | 38.8 c | 4.0 b | 3.9 b |
| CV(%) | 5.7 | 11.5 | 4.2 | 11.4 | 7.2 | 6.1 |
| 2014 |  |  |  |  |  |  |
| LS | 41.0 b | 36.4 c | 52.0 a | 54.5 a | 5.7 a | 5.6 a |
| MS | 42.3 b | 42.4 b | 50.5 a | 53.0 b | 5.3 ab | 5.2 ab |
| HS | 46.6 a | 46.3 a | 49.2 b | 41.3 c | 5.1 b | 4.9 b |
| CV(%) | 5.7 | 11.9 | 2.8 | 14.6 | 5.7 | 6.7 |

LS, low soil salinity; MS, medium soil salinity; HS, high soil salinity.

Sucrose transformation rate (%) was calculated as 100[(maximum sucrose content − minimum sucrose content)/maximum sucrose content].

Values followed by different letters within the same column are significantly different at a probability level of *P*=0.05. Each value represents the mean of three replications. * and ** indicate significant differences at 0.05 and 0.01 probability levels, respectively.
