# Supplementary material for: Effects of Soil Salinity on Sucrose Metabolism in Cotton Leaves
Source: PLoS One. 2016 May 26;11(5):e0156241. doi: 10.1371/journal.pone.0156241 (PMC4881904; doi:10.1371/journal.pone.0156241)
Supplement: S2 Table — LS: low soil-salinity, MS: medium soil-salinity, and HS: high soil-salinity. (DOCX) [file pone.0156241.s002.docx]

**S2 Table. The peak values of sucrose phosphate synthase (SPS) and sucrose synthase (SuSy) activities in the youngest fully expanded main-stem leaf (MSL) and the subtending leaf of cotton boll (LSCB) under different soil salinity levels in 2013 and 2014.**

| Years | Salinity levels | SPS activity (mg Sucrose g^-1^ FW h^-1^) | | SuSy activity (mg Sucrose g^-1^ FW h^-1^) | |
| --- | --- | --- | --- | --- | --- |
|  |  | CCRI-79 | Simian 3 | CCRI-79 | Simian 3 |
| MSL |  |  |  |  |  |
| 2013 | LS | 7.36 a | 8.06 a | 10.93 a | 9.95 a |
|  | MS | 7.66 a | 9.94 b | 11.61 ab | 10.95 b |
|  | HS | 8.04 b | 10.44 c | 12.98 b | 12.04 c |
|  | CV(%) | 4.5 | 13.3 | 8.9 | 9.5 |
| 2014 | LS | 6.80 a | 5.79 a | 8.63 a | 8.91 a |
|  | MS | 7.21 ab | 6.50 b | 9.30 b | 9.88 b |
|  | HS | 7.46 b | 8.02 c | 9.74 b | 10.63 c |
|  | CV(%) | 4.7 | 16.8 | 6.1 | 8.8 |
| LSCB |  |  |  |  |  |
| 2013 | LS | 7.35 a | 7.07 a | 8.14 a | 5.69 a |
|  | MS | 8.08 b | 10.08 b | 8.98 b | 6.51 b |
|  | HS | 8.58 b | 11.98 c | 10.03 c | 7.13 c |
|  | CV(%) | 7.8 | 25.5 | 10.5 | 11.3 |
| 2014 | LS | 8.89 a | 8.17 a | 6.98 a | 8.19 a |
|  | MS | 10.28 b | 10.02 b | 8.73 b | 9.52 b |
|  | HS | 10.39 b | 12.34 c | 9.36 c | 10.98 c |
|  | CV(%) | 8.5 | 20.5 | 14.8 | 14.6 |

LS, low soil salinity; MS, medium soil salinity; HS, high soil salinity.

Values followed by a different lowercase letter within the same column indicate significant differences at a probability level of *P* = 0.05.
